# Attention

**Published:** 1883-10

**Authors:** 


### ATTENTION!

This number of the INDEPENDENT PRACTITIONER contains sixty pages; which makes it considerably larger than the average dental journal. We shall continue to enlarge it as the support afforded it will warrant, and hope that next year we shall be enabled to afford sixty-four pages to each number.

